# Supplementary material for: Evaluation of VIDAS® Diagnostic Assay Prototypes Detecting Dengue Virus NS1 Antigen and Anti-Dengue Virus IgM and IgG Antibodies
Source: Diagnostics (Basel). 2021 Jul 7;11(7):1228. doi: 10.3390/diagnostics11071228 (PMC8307080; doi:10.3390/diagnostics11071228)
Supplement: Supplementary file 1 [file diagnostics-11-01228-s001.zip › diagnostics-1265891-SI.pdf]

Supplementary Tables

Table S1. Positive agreement of the respective NS1 antigen assays with RT-PCR calculated on the common cohort (N=55)

Reference test	Population	VIDAS		FOCUS		RDT[a]	
		n/N	% [95% CI]	n/N	% [95% CI]	n/N	% [95% CI]
RT-PCR	Total	47/55	85.5% [73.8-92.4]	46/55	83.6% [71.7-91.1]	40/55	72.7% [59.8-82.7]
	Adults	30/38	78.9% [63.7-88.9]	30/38	78.9% [63.7-88.9]	24/38	63.2% [47.3-76.6]
	Children	17/17	100.0% [80.5-100.0]	16/17	94.1% [73.0-99.0]	16/17	94.1% [73.0-99.0]

[a]SD BIOLINE Dengue Duo Rapid Test (Dengue NS1 Ag); n/N is the ratio of the number of samples positive for the respective immunoassays (VIDAS®, FOCUS, RDT) to the number of RT-PCR-positive samples; Fisher's Exact Test between adults and children: VIDAS® (p=0.048), FOCUS (p=0.247) and RDT (p=0.022); McNemar VIDAS® vs. Focus (p=0.564); McNemar VIDAS® vs. RDT (p=0.008); Abbreviations: CI, confidence interval; RDT, rapid diagnostic test.

Table S2. Concordance of VIDAS® Dengue NS1 Ag and competitor assays calculated on the common cohort (N=89)

Reference test	Population	Positive Agreement		Negative Agreement		Overall Agreement	
		n/N	% [95% CI]	n/N	% [95% CI]	n/N	% [95% CI]
Dengue NS1 Antigen DxSelect™ (Focus)	Total	78/80	97.5% [91.3-99.7]	7/9	77.8% [45.3-93.7]	85/89	95.5% [88.9-98.8]
	Adults	47/48	97.9% [88.9-99.9]	7/8	87.5% [52.9-97.8]	54/56	96.4% [87.7-99.6]
	Children	31/32	96.9% [83.8-99.9]	0/1	0.0% [0.0-97.5]	31/33	93.9% [80.4-98.3]
NS1 RDT[a]	Total	70/70	100.0% [94.9-100.0]	9/19	47.4% [27.3-68.3]	79/89	88.8% [80.5-93.8]
	Adults	41/41	100.0% [91.4-100.0]	8/15	53.3% [30.1-75.2]	49/56	87.5% [76.4-93.8]
	Children	29/29	100.0% [88.1-100.0]	1/4	25.0% [4.6-69.9]	30/33	90.9% [76.4-96.9]

[a]SD BIOLINE Dengue Duo Rapid Test (Dengue NS1 Ag); n/N is the ratio of the number of VIDAS® assays in agreement with the competitor assays (Reference test) to the total number of competitor assays (positive, negative and overall, respectively); Fisher's Exact Test between adults and children on overall agreement: VIDAS® vs. FOCUS (p=0.625) and VIDAS® vs. RDT (p=0.739); Abbreviations: CI, confidence interval; RDT, rapid diagnostic test.

Table S3. Concordance of VIDAS® Anti-Dengue IgM and competitor assays calculated on the common cohort (N=83)

Reference test	Population	Positive Agreement		Negative Agreement		Overall Agreement	
		n/N	% [95% CI]	n/N	% [95% CI]	n/N	% [95% CI]
Panbio Dengue IgM Capture ELISA	Total	38/45	84.4% [71.2-92.3]	24/38	63.2% [47.3-76.6]	62/83	74.7% [64.4-82.8]
	Adults	24/29	82.8% [65.5-92.4]	15/24	62.5% [42.7-78.8]	39/53	73.6% [60.4-83.6]
	Children	14/16	87.5% [64.0-96.5]	9/14	64.3% [38.8-83.7]	23/30	76.7% [59.1-88.2]
IgM RDT[a]	Total	31/36	86.1% [71.3-93.9]	26/47	55.3% [41.2-68.6]	57/83	68.7% [58.1-77.6]
	Adults	21/25	84.0% [65.3-93.6]	16/28	57.1% [39.1-73.5]	37/53	69.8% [56.5-80.5]
	Children	10/11	90.9% [62.3-98.4]	10/19	52.6% [31.7-72.7]	20/30	66.7% [48.8-80.8]

[a]SD BIOLINE Dengue Duo Rapid Test (Dengue IgG/IgM); n/N is the ratio of the number of VIDAS® assays in agreement with the competitor assays (Reference test) to the total number of competitor assays (positive, negative and overall, respectively); Chi-square Test between adults and children on overall agreement: VIDAS® vs. Panbio (p=0.756) and VIDAS® vs. RDT (p=0.767); Abbreviations: CI, confidence interval; RDT, rapid diagnostic test.

Table S4. Concordance of VIDAS® Anti-Dengue IgG and competitor assays calculated on the common cohort (N=83)

Reference test	Population	Positive Agreement		Negative Agreement		Overall Agreement	
		n/N	% [95% CI]	n/N	% [95% CI]	n/N	% [95% CI]
Panbio Dengue IgG Indirect ELISA	Total	51/67	76.1% [64.7-84.7]	12/16	75.0% [50.5-89.8]	63/83	75.9% [65.7-83.8]
	Adults	34/43	79.1% [64.8-88.6]	7/10	70.0% [39.7-89.2]	41/53	77.4% [64.5-86.5]
	Children	17/24	70.8% [50.8-85.1]	5/6	83.3% [43.6-97.0]	22/30	73.3% [55.6-85.8]
IgG RDT[a]	Total	33/37	89.2% [75.3-95.7]	24/46	52.2% [38.1-65.9]	57/83	68.7% [58.1-77.6]
	Adults	27/29	93.1% [78.0-98.1]	14/24	58.3% [38.8-75.5]	41/53	77.4% [64.5-86.5]
	Children	6/8	75.0% [40.9-92.9]	10/22	45.5% [26.9-65.3]	16/30	53.3% [36.1-69.8]

[a]SD BIOLINE Dengue Duo Rapid Test (Dengue IgG/IgM); n/N is the ratio of the number of VIDAS® assays in agreement with the competitor assays (Reference test) to the total number of competitor assays (positive, negative and overall, respectively); Chi-square Test between adults and children on overall agreement: VIDAS® vs. Panbio (p=0.680) and VIDAS® vs. RDT (p=0.023); Abbreviations: CI, confidence interval; RDT, rapid diagnostic test.
